# Retraction: Monoclonal IgG antibodies generated from joint-derived B cells of RA patients have a strong bias toward citrullinated autoantigen recognition

**DOI:** 10.1084/jem.2012148612112018r

**Published:** 2019-01-07

**Authors:** Khaled Amara, Johanna Steen, Fiona Murray, Henner Morbach, Blanca M. Fernandez-Rodriguez, Vijay Joshua, Marianne Engström, Omri Snir, Lena Israelsson, Anca I. Catrina, Hedda Wardemann, Davide Corti, Eric Meffre, Lars Klareskog, Vivianne Malmström

Vol. 210, No. 3, March 11, 2013. 10.1084/jem.20121486

The editors of the *Journal of Experimental Medicine* have been notified by Dr. Vivianne Malmström of the Karolinska University Hospital, Karolinska Institutet, Stockholm, Sweden, that she and the other authors of the above article wish to retract the paper.

The authors state:

In our efforts to understand the contribution of autoreactive B cells and autoantibodies in rheumatoid arthritis (RA), we have continued to drive projects where we study B cells and plasma cells from different anatomical compartments and express patient-derived recombinant mAbs for downstream applications. Some of these efforts have recently been published (e.g., Steen, J., et al. 2018. *Arthritis Rheumatol.* https://doi.org/10.1002/art.40699). However, when using the mAbs first described in the above article as comparators to the newly generated mAbs in affinity measurements, we observed that the *K*_d_ values from our study could not be reproduced. After reviewing the original SPR data, we learned that the discrepancy was due to an incorrect instrument setting, and we can now conclude that the mAbs in our paper have no measurable affinity for the tested citrullinated peptides, while a number of our new mAbs do.

Moreover, although we initially used widely spread protocols for both the purification of recombinantly expressed mAbs and the subsequent testing of their reactivity in ELISA, we grew aware that these protocols were not fully optimized for our research setting. Hence, in the time between the above article and the follow-up studies, we have continuously improved our methods and significantly refined our protocols based on state-of-the-art recombinant antibody methodology. We believe that this is critical for studies of antibodies from patients with pronounced autoreactivity. Caveats include storage of antibodies, aggregation tests, and excluding unspecific so-called polyreactivity (generally driven by charge or hydrophobic interaction). So, although the antibodies presented in the above article were positive based on published methodologies and the cut-offs used at the time, today we no longer regard them as citrulline-specific autoantibodies based on subsequent findings in our follow-up studies. The precise adjustments of the protocols to allow the distinction of sticky/polyreactive versus antigen-specific autoantibodies will be presented elsewhere.

Notably, mAbs from the above article still represent Ig sequences generated from the RA joint with interesting biochemical characteristics, which may provide insights into joint pathology. Indeed, some of them demonstrate the capacity to functionally contribute to disease by mechanisms that we continue to investigate.

The authors wish to communicate their apologies for their mistake concerning the SPR measurements and the misinterpretation of the ELISA data reported in the paper.

